# A Rare Case Report of a Human *Dirofilaria repens* Infection

**DOI:** 10.3390/microorganisms13030476

**Published:** 2025-02-21

**Authors:** Christoph Schatz, Magdalena Füßl, Yasemin Caf, Katja Schmitz, Daniela Kresse, Wilhelm Ludwig, Julia Walochnik, Ludwig Knabl

**Affiliations:** 1 Tyrolpath Obrist Brunhuber GmbH, Hauptplatz 4, 6511 Zams, Austria; 2 University Hospital for Inner Medicine II, Tirol Kliniken, Anichstraße 35, 6020 Innsbruck, Austria; 3 Institute of Specific Prophylaxis and Tropical Medicine, Medical University of Vienna, 1090 Wien, Austria

**Keywords:** *Dirofilaria repens*, parasitology, molecular diagnostics

## Abstract

In June 2024, a 41 year-old woman presented to the infectious diseases outpatient clinic with a left inguinal mass progressing in size. The patient had previously been on vacation in Greece. When a tumor was initially suspected, the mass was surgically removed. Staining with Grocott methenamine silver and Alzian blue were inconspicuous, but histopathologic examination revealed a clear histiocytic demarcation, followed by a confirmation of the suspected diagnosis of dirofilariasis caused by *Dirofilaria repens* by PCR. Even though still a rare event in Austria, the number of human *D. repens* cases has been continuously increasing in recent years. This is partly due to the increased spread of the parasite due to climate change and globalization.

## 1. Introduction

The filaroid nematode *Dirofilaria repens* is endemic in Asia [1,2], Africa, and large parts of southern Europe [3,4], especially in Italy [5], southern France, and Greece. The prevalence of *D. repens* has increased in many areas and in the recent past autochthonous cases have been reported from several central and northern European countries [4,6], including Serbia [7], Poland [8], and France [9]. In addition to dogs, also foxes, cats, ferrets, raccoons, bears [4,10], and humans can be infected. *D. repens* is a parasite of domestic and wild canids [6] transmitted as third-stage larvae (L3) by mosquitos (Culicidae), including various species of the genera *Aedimorphus*, *Anopheles*, *Armigeres*, *Ochlerotatus*, *Stegomyia*, *Culex*, *Coquillettidia*, and *Mansonia* [10,11]. Humans are usually dead-end hosts, resulting in an incomplete last developmental cycle of *D. repens*. In very rare cases, however, microfilariasis can also occur, in which humans serve as the final hosts [7,8,9]. Despite the rarity of microfilariasis in humans, cases have been described in Serbia, Poland, and France, in which the *D. repens* infection manifested itself as microfilarasis. Regarding the symptoms that the parasite causes in humans, *D. repens* typically affects the skin [2] and the eyes [12]. While skin manifestation is characterized by subcutaneous and cutaneous nodules, which are triggered by dwelling pre-adult and adult parasites, ocular dirofilariasis is characterized by subconjunctival, intravitreal, or intraorbital infections, manifesting as inflammatory reactions, visual disturbances, and potential vision loss due to the presence and migration of adult worms in various ocular and periocular tissues. In contrast, pulmonary, genital, or mammary lesions occur infrequently. Additionally, migrating larvae and (im)mature worms may cause ascites, hydropericardium, and lymphadenomegaly with granulomatous or eosinophilic dermatitis [2].

Dogs are the main reservoir hosts for *D. repens*, but a wide range of other canids are also known to be suitable hosts. The life cycle of *D. repens* includes four larval stages with a total development time of 6–9 months [13]. Infected mosquitos function as carriers and deliver L3 larvae through the skin during a blood meal, which the mosquitos themselves ingested by biting infected mammals [13]. Larvae develop from the L1 stage to the L2 stage in the digestive tract of the mosquito, and in an additional 3 days the larvae reach the L3 stage [13]. The larvae are transmitted through the bite of an infected mosquito and develop into adult worms in dogs and other canids. These adult worms produce microfilariae that enter the bloodstream, which is called microfilariasis [14]. In the accidental host human host, *D. repens* usually reside within subcutaneous nodules as immature worms. Further development into mating worms is rare, and the resulting microfilariasis is even rarer [14].

Regarding the latter, it is also being discussed whether *D. repens* can adapt to humans as hosts. Although it was previously assumed that humans are only intermediate hosts for this parasite, the formation of microfilariae and their release into the systemic circulation in humans indicate that humans may also act as final hosts for *D. repens* [7].

Treatment of *D. repens* infection usually involves surgical removal of the parasite, with additional treatment with anthelmintics or antibiotics in some cases [3].

## 2. Case Description

**Figure 1:**
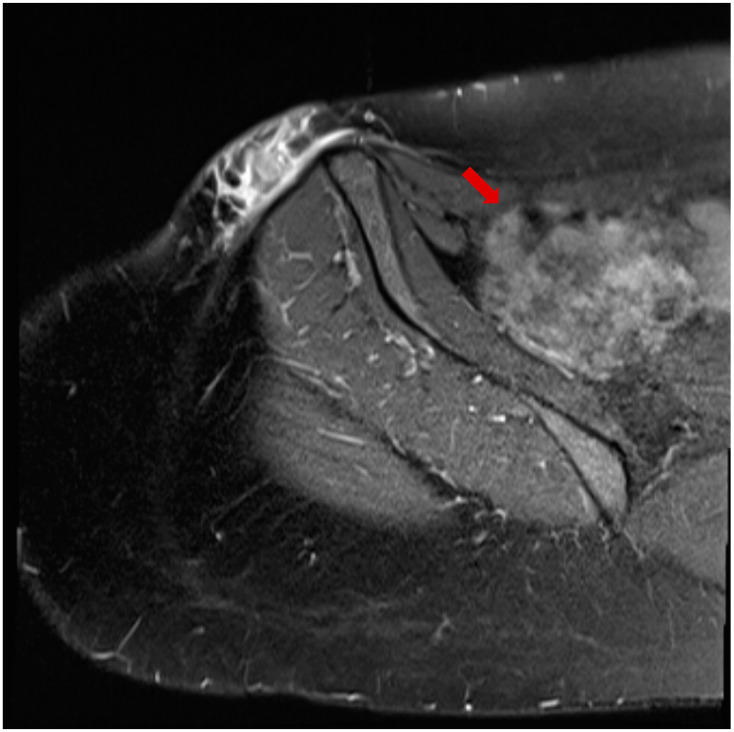
MRT image of the swelling in the left groin showing a mass measuring 3.9 cm in length, 2.6 cm in width, and 1.5 cm in depth (arrow).

**Figure 2:**
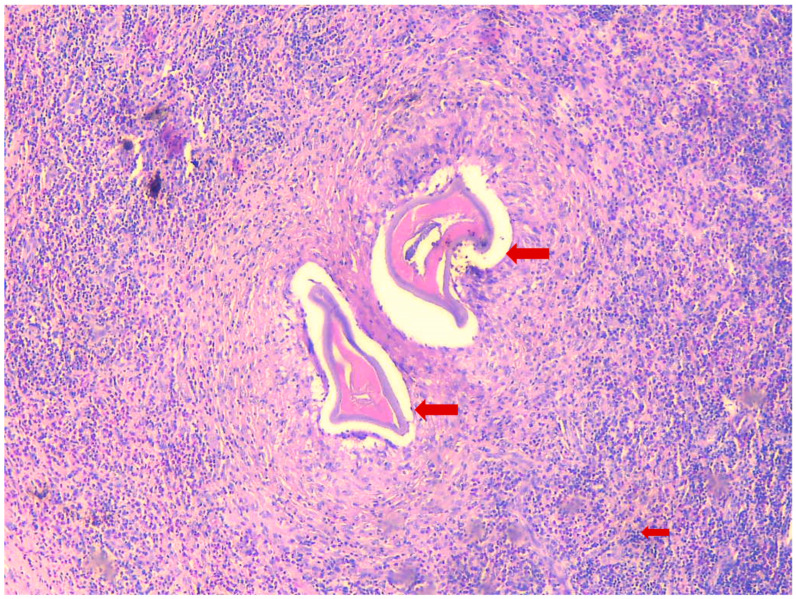
Histopathological section of the biopsy, H&E staining, 20×, a histopathologic section of the biopsy with a granuloma and cross sections of dirofilarial; Small arrow shows the inflammatory infiltrate with lymphocytes and eosinophils; Large arrows show cross sections of the worm.

The patient, a 41-year-old female, presented to the infectious diseases outpatient clinic in June 2024 after noticing a swelling in the left groin area that had progressed in size. The first symptoms appeared in April 2024. Initially, the swelling resembled a mosquito bite and did not attract the patient’s attention. But the subcutaneous nodule persisted and began to grow steadily over the course of 8 weeks. During the anamnesis, the patient stated that she often traveled to Kefalonia in Greece. Her last trip before the medical clarification and excision was in August 2023. Symptoms were relatively mild and limited to mild itching at the site of the swelling. The patient reported no systemic symptoms and stated that she was in good physical condition. There were no relevant prior diseases, except a mild iron deficiency syndrome. The complete blood count was unremarkable, but the differential blood count revealed a slight eosinophilia (407 cells/µL and 9.2%, respectively). Three consecutive stool analyses for parasites were negative. Ultrasound examination revealed the swelling to be an encapsulated, easily displaceable mass measuring 3.9 cm in length, 2.6 cm in width, and 1.5 cm in depth. The mass was partially filled with fluid. The MRI confirmed the result of the sonographic examination, with the growing mass being diagnosed as an unclear tumor (Figure 1). It was decided to surgically remove the mass in June 2024 and to send the resected tissue to a pathology laboratory for further examination. On macroscopic examination, the tumor presented as a nodular, beige-colored mass with a lipomatous character. The resected subcutaneous adipose tissue presented with panniculitis and focal abscess formation. There was a clear histiocytic demarcation from the tumor mass, which had a chitin-like character and a cystic configuration (Figure 2). The resected tissue did not stain with Grocott methenamine silver, Alzian blue, or Giemsa. Only Periodic acid-Schiff (PAS) staining showed a delicate colorability of the tumor material. In sum, the histopathological examination results strongly suggested a parasitic infestation. Formalin-fixed paraffin-embedded tumor material was transferred to the parasitology lab at the Medical University of Vienna for further clarification. Based on the sections, the symptoms, and the travel history, the presumptive diagnosis dirofilariasis was made, which was proven by a polymerase chain reaction (PCR) specific for *D. repens* [15] after DNA extraction from the fixed material. The patient was completely free of symptoms after removal of the mass, with simultaneous regression of eosinophilia, thus no antiparasitic therapy was initiated. Abdominal ultrasonography and exploratory echocardiography performed in September 2024 during a follow-up examination of the patient were unremarkable, so there was no evidence of organ involvement.

## 3. Discussion

While *D. repens* infections occur frequently in dogs, which in endemic areas are the definitive hosts of this parasite and show subcutaneous nodules, diffuse dermatitis, skin lesions, or more specific skin manifestations such as erythematous scaling, human infestations are comparably rare [16]. In a study from 2005, microfilariae of *D. repens* were detected in 34.5% of all investigated dogs in Slovakia [17], a neighboring country of Austria. Thus far, twelve cases of human *D. repens* infections have occurred between 2007 and 2017 in Slovakia [18].

In Greece, during a screening of dogs, it was found that 2.3% of the 750 examined animals had *D. repens* microfilariasis [19]. From early 2015 until May 2021, 46 cases of *D. repens* infections in humans were detected in the Balkan region [20]. In Austria, 39 cases of human dirofilariasis have been reported between 1978 and 2020, generally increasing since 1998 [21], whereby also autochthonous cases of *D. repens* infections in humans have occurred [22]. In general, the incidence in Austria has increased considerably [21]. A sero-epidemiological study screening dogs for antibodies against *Dirofilaria* spp. revealed a positivity rate of 3%, which is higher than the mean of 2.6% of infected dogs over all eight countries in Central Europe and the UK [23]. *D. repens* DNA was detected in mosquitos in eastern Austria in 2012 [24], which could lead to a higher number of infections in future, as the detection of the parasite in domestic mosquitos can be interpreted as the first step towards Austria becoming an endemic area.

There are indications that *D. repens* is spreading more rapidly from the endemic areas in southern Europe towards the north than *D. immitis* [6]. For example, *D. repens* infections have already been described in dogs in Estonia [25]. It is suspected that the increasing number of infected dogs is due to increased travel activity, increased taking of dogs with travelers, and the increasing adoption of dogs from endemic areas [26]. Dogs can serve as transmission vectors for humans, leading to the spread of the parasite through infected dogs, with the associated infections in humans in areas where the parasite was not previously native. The reasons for the parasite’s northward spread include global warming and globalization, which have resulted in an expansion of the mosquito vector range, as well as the importation of pets from and the travel of pets to southern countries [6].

In the current case, the infection assumedly was acquired in an epidemic region in Greece during a stay in August 2023 and resulted in the excision of a non-small tumor in June 2024. Thus, the incubation period from infection to the development of noticeable symptoms was several months, which is consistent with the incubation period of other described cases. Nevertheless, there have also been cases where the time between infection and the development of *D. repens*-associated symptoms was shorter. In one case from Croatia, for example, an incubation period of 7 months was described [27]. Of course, the possibility of an autochthonous infection with the parasite in Austria cannot be completely ruled out. In the past, autochthonous *D. repens* cases have been described from Austria, although the infections always occurred in the east of the country and not, as in the current case, in Tyrol, which is located in western Austria. In 2008, a 34 year-old woman was bitten by a mosquito at the Austrian-Hungarian border, whereupon she later developed symptoms of a subcutaneous *D. repens* infection, representing the first documented case of a *D. repens* infection acquired in Austria [28]. In a more recent case, a woman became infected in 2022 after a mosquito bite in Vienna during a visit to the Lobau, a floodplain in the south-west of Vienna, and developed a subcutaneous nodule that was identified as *D. repens* [22].

Although suffering from a manifest parasitic infection, the patient in the present case did not show any pronounced eosinophilia. This phenomenon has already been described in the literature [22]. However, in other patients eosinophil granulocytes comprised up to 48% of the leukocytes [3]. But these were patients with microfilaremia, which is associated with a significantly stronger immune response compared to an encapsulated worm. In addition to the mild eosinophilia in our patient, she did not suffer from any systemic signs such as fatigue or fever. For this reason, only surgical removal without concomitant antihelminthic therapy was performed in the present patient, which ultimately led to a complete cure. If, on the other hand, microfilaremia occurs, drug therapy is essential. The use of doxycycline, for example, has proven effective here. Doxycycline attacks the bacterial endosymbiont of *D. repens*, *Wolbachia*, and leads to a long-term embryostatic effect and the sterility of the female worms [14]. This sustainably reduces the larvae in the bloodstream or the lymphatic system [29,30].

The role of *Wolbachia* in *Dirofilaria* species has been studied since the 1970s, when Harada and colleagues found bacterium-like bodies in filariae using electron microscopy [31]. Subsequent investigations were able to assign these particles to the order *Ricksettiales* and the genus *Wolbachia* [32]. It was shown that *Wolbachia* inhibits the apoptosis of germ-line cells and the embryos of *D. repens* and provides essential metabolites for the development and regulation of genes of *D. repens* that are important for embryogenesis and the molting of the parasite. Although the exact background of the symbiotic relationship is not yet fully understood, it is assumed that the secretion of proteins such as the Wolbachia surface protein (WSP) leads to the inhibition of apoptosis. *Wolbachia* spp. provide important metabolites, for example, from the heme biosynthetic pathway, which *D. repens* lacks. As an iron chelator, heme protects against oxidative stress. In addition, it is discussed whether *Wolbachia* possibly provides enzymes for nucleotide-, fatty acid-, and folic acid synthesis [33].

For this reason, doxycycline, which kills the endosymbiotic bacteria, is a good treatment option in dirofilariasis. In veterinary medicine, both doxycycline and macrocyclic lactones, such as moxidectin, are used to treat *D. repens* infections, which lead to synergistic effects and increases the adulticidal efficacy [34].

However, as our patient did not suffer from microfilaremia and it was assumed that the infection with *D. repens* was limited to the inguinal mass, only the surgical removal of the parasite was performed without concomitant antibiotic or antihelminthic therapy.

The current case highlights the importance of considering parasitic infections during the diagnostic process also in central European countries like Austria. Due to the steady increase in travel and global warming, which particularly favors the spread of these vector-borne diseases, it must be expected that the prevalence of dirofilariasis will continue to increase in Central Europe.

## Data Availability

The original contributions presented in this study are included in the article. Further inquiries can be directed to the corresponding author.
